# Poly[[μ_2_-1,4-bis­(imidazol-1-ylmeth­yl)benzene]bis­(μ_4_-cyclo­hexane-1,4-dicarboxyl­ato)dicobalt(II)]

**DOI:** 10.1107/S160053680903428X

**Published:** 2009-09-05

**Authors:** Qun-Di Yu, Da-Jun Sun, Edward R. T. Tiekink

**Affiliations:** a Food Science and Pharmacy College, Zhejiang Ocean University, Zhoushan 316000, People’s Republic of China; b Department of Vascular Surgery, the China–Japan Union Hospital of Jilin University, Changchun, 130033, People’s Republic of China; c Department of Chemistry, Universidade Federal de São Carlos, 13565-905 São Carlos, SP, Brazil

## Abstract

In the title compound, [Co_2_(C_8_H_10_O_4_)_2_(C_14_H_14_N_4_)]_*n*_, the two Co^II^ atoms are both five-coordinated by four carboxyl­ate O atoms, derived from two different cyclo­hexane-1,4-dicarboxyl­ate (chdc) ligands, and an N atom, derived from one end of a 1,4-bis­(imidazol-1-ylmeth­yl)benzene mol­ecule (1,4-bix), in a distorted square-pyramidal environment. Each end of the chdc ligand links pairs of Co^II^ atoms into a paddle-wheel assembly, *i.e.* Co_2_(O_2_C*R*′)_4_; these are connected into rows because of the bridging nature of the chdc ligands, and the rows are further connected into a two-dimensional layer through the 1,4-bix ligands. The 1,4-bix ligand, which is disposed about a centre of inversion, is disorderd. Two positions were discerned for the –CH_2_(C_6_H_4_)CH_2_– residue, with the major component having a site-occupancy factor of 0.512 (9).

## Related literature

For background to coordination polymers, see: Yang *et al.* (2008 ▶). For the isotypic Ni(II) structure, see: Li *et al.* (2009 ▶).
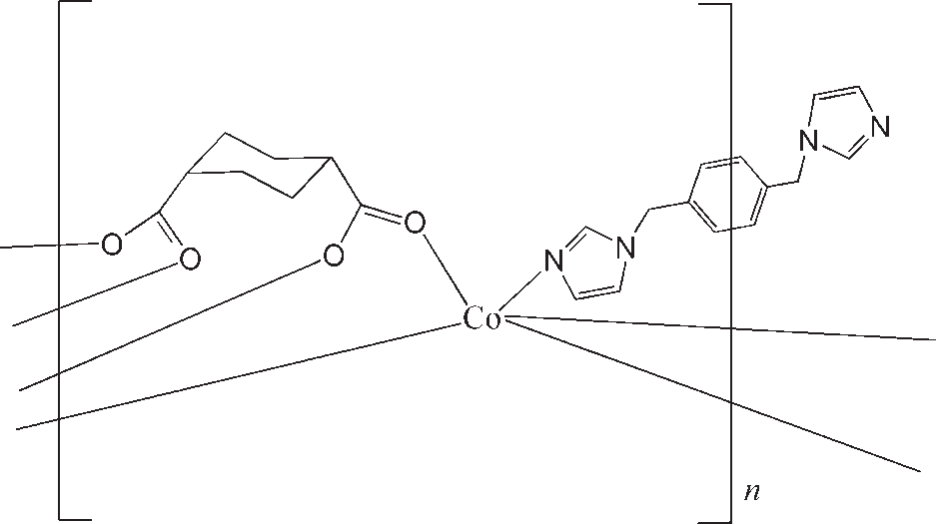


## Experimental

### 

#### Crystal data

[Co_2_(C_8_H_10_O_4_)_2_(C_14_H_14_N_4_)]
*M* *_r_* = 696.48
Triclinic, 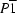

*a* = 8.5415 (6) Å
*b* = 8.8051 (5) Å
*c* = 10.8007 (5) Å
α = 93.824 (4)°
β = 100.940 (4)°
γ = 105.413 (5)°
*V* = 762.95 (8) Å^3^
*Z* = 1
Mo *K*α radiation
μ = 1.14 mm^−1^
*T* = 293 K
0.24 × 0.22 × 0.21 mm

#### Data collection

Bruker APEX diffractometer
Absorption correction: multi-scan (*SADABS*; Sheldrick, 1996 ▶) *T*_min_ = 0.756, *T*_max_ = 0.788
6296 measured reflections
2663 independent reflections
2212 reflections with *I* > 2σ(*I*)
*R*_int_ = 0.030

#### Refinement

*R*[*F* ^2^ > 2σ(*F* ^2^)] = 0.052
*wR*(*F* ^2^) = 0.150
*S* = 1.06
2663 reflections
206 parameters
30 restraints
H-atom parameters constrained
Δρ_max_ = 1.34 e Å^−3^
Δρ_min_ = −1.40 e Å^−3^

### 

Data collection: *SMART* (Bruker, 1998 ▶); cell refinement: *SAINT* (Bruker, 1998 ▶); data reduction: *SAINT*; program(s) used to solve structure: *SHELXS97* (Sheldrick, 2008 ▶); program(s) used to refine structure: *SHELXL97* (Sheldrick, 2008 ▶); molecular graphics: *SHELXTL* (Sheldrick, 2008 ▶); software used to prepare material for publication: *SHELXTL*.

## Supplementary Material

Crystal structure: contains datablocks global, I. DOI: 10.1107/S160053680903428X/bt5034sup1.cif

Structure factors: contains datablocks I. DOI: 10.1107/S160053680903428X/bt5034Isup2.hkl

Additional supplementary materials:  crystallographic information; 3D view; checkCIF report

## Figures and Tables

**Fig. 1.**
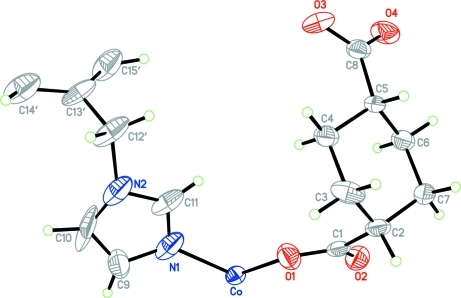
The asymmetric unit in the polymeric structure of (I), showing the atomic numbering scheme. Displacement ellipsoids are drawn at the 30% probability level. Only one component of the disordered -CH2(C6H4)CH2- residue is shown for reasons of clarity.

**Fig. 2.**
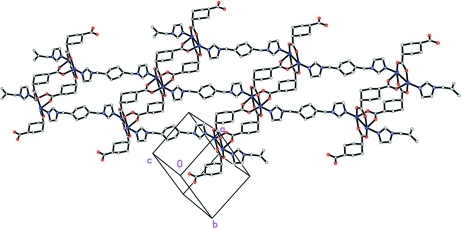
View of the two-dimensional layer in (I). H atoms have been omitted for clarity.

## supplementary crystallographic information

### Comment

So far, the rigid N-containing bridging ligand, such as 4,4'-bipyridine, has been widely used in the construction of metal-organic polymers, however, the flexible N-donor ligand, such as 1,4-bis(imidazol-1-ylmethyl)benzene (1,4-bix), has not been well studied (Yang *et al.*, 2008). In this work, 1,4-bix assembles with cobalt cyclohexane-1,4-dicarboxylate (chdc) to give a two-dimensional polymer [Co_2_(chdc)_2_(1,4-bix)] (I).

The compound (I) is isostructural with reported Ni(II) compound (Li *et al.*, 2009). The asymmetric unit of (I) comprises a Co atom, a chdc dianion, and half a 1, 4-bix molecule which is disposed about a centre of inversion (Fig. 1). Each Co^II^ atom is five-coordinated by four carboxylate O atoms, derived from two different chdc ligands, and an N atom, derived from one end of a 1,4-bix molecule, in distorted square pyramidal sphere. Each end of the chdc ligand links pairs of Co^II^ atoms into a paddle-wheel assembly, *i.e.* Co_2_(O_2_CR')_4_. These are connected into rows because of the bridging nature of the chdc ligands, and rows are further connected into a two-dimensional layer through the 1,4-bix ligands. If the second Co atom in the paddle-wheel assembly is considered as occupying a coordination site, the Co···Co distance is 2.721 (6) Å, the coordination geometry would be distorted octahedral.

### Experimental

H_2_chdc (0.5 mmol), 1,4-bix (0.5 mmol) and cobalt chloride hexahydrate (0.5 mmol) were placed in water (15 ml), and triethylamine was added until the pH value of the solution was 5.6. The solution was heated in a 23 ml Teflon-lined stainless-steel autoclave at 445 K for 3 days. The autoclave was cooled to room temperature over several hours. Purple crystals were isolated in about 52% yield.

### Refinement

Carbon-bound H-atoms were placed in calculated positions with C—H = 0.93 - 0.98 Å, and were included in the refinement in the riding model approximation, with *U*(H) set to 1.2*U*_eq_(C).

Disorder was noted in bridging 1,4-bix ligand. Two positions were discerned for the -H_2_C(C_6_H_4)_CH_2_- residue. From refinement, the major component had a site occupancy of 0.512 (9). Multiple positions were not resolved for the imidazole ring, even though several of the atoms exhibited elongated displacement ellipsoids. The atoms of this ring were restrained to be approximately isotropic with application of the ISOR command in SHELXL-97 (Sheldrick, 2008).

The maximum and minimum residual electron density peaks of 1.34 and -1.40 eÅ^-3^, respectively, were located 0.08 Å and 0.05 Å from the N1 and N2 atoms, respectively.

### Crystal data

**Table:**

[Co_2_(C_8_H_10_O_4_)_2_(C_14_H_14_N_4_)]	*Z* = 1
*M**_r_* = 696.48	*F*(000) = 360
Triclinic, *P*1	*D*_x_ = 1.516 Mg m^−^^3^
Hall symbol: -P 1	Mo *K*α radiation, λ = 0.71073 Å
*a* = 8.5415 (6) Å	Cell parameters from 2663 reflections
*b* = 8.8051 (5) Å	θ = 3.0–25.0°
*c* = 10.8007 (5) Å	µ = 1.14 mm^−^^1^
α = 93.824 (4)°	*T* = 293 K
β = 100.940 (4)°	Block, purple
γ = 105.413 (5)°	0.24 × 0.22 × 0.21 mm
*V* = 762.95 (8) Å^3^	

### Data collection

**Table:**

Bruker APEX diffractometer	2663 independent reflections
Radiation source: fine-focus sealed tube	2212 reflections with *I* > 2σ(*I*)
graphite	*R*_int_ = 0.030
φ and ω scans	θ_max_ = 25.0°, θ_min_ = 4.3°
Absorption correction: multi-scan (*SADABS*; Sheldrick, 1996)	*h* = −9→10
*T*_min_ = 0.756, *T*_max_ = 0.788	*k* = −10→10
6296 measured reflections	*l* = −12→12

### Refinement

**Table:**

Refinement on *F*^2^	Primary atom site location: structure-invariant direct methods
Least-squares matrix: full	Secondary atom site location: difference Fourier map
*R*[*F*^2^ > 2σ(*F*^2^)] = 0.052	Hydrogen site location: inferred from neighbouring sites
*wR*(*F*^2^) = 0.150	H-atom parameters constrained
*S* = 1.06	*w* = 1/[σ^2^(*F*_o_^2^) + (0.0864*P*)^2^ + 1.1188*P*] where *P* = (*F*_o_^2^ + 2*F*_c_^2^)/3
2663 reflections	(Δ/σ)_max_ = 0.001
206 parameters	Δρ_max_ = 1.34 e Å^−^^3^
30 restraints	Δρ_min_ = −1.40 e Å^−^^3^

### Special details

**Table:**

Geometry. All s.u.'s (except the s.u. in the dihedral angle between two l.s. planes) are estimated using the full covariance matrix. The cell s.u.'s are taken into account individually in the estimation of s.u.'s in distances, angles and torsion angles; correlations between s.u.'s in cell parameters are only used when they are defined by crystal symmetry. An approximate (isotropic) treatment of cell s.u.'s is used for estimating s.u.'s involving l.s. planes.
Refinement. Refinement of F^2^ against ALL reflections. The weighted R-factor wR and goodness of fit S are based on F^2^, conventional R-factors R are based on F, with F set to zero for negative F^2^. The threshold expression of F^2^ > 2σ(F^2^) is used only for calculating R-factors(gt) etc. and is not relevant to the choice of reflections for refinement. R-factors based on F^2^ are statistically about twice as large as those based on F, and R- factors based on ALL data will be even larger.

### Fractional atomic coordinates and isotropic or equivalent isotropic displacement parameters (Å^2^)

**Table:**

	*x*	*y*	*z*	*U*_iso_*/*U*_eq_	Occ. (<1)
Co	1.02761 (7)	0.46504 (6)	0.62430 (5)	0.0287 (2)	
O1	0.9612 (4)	0.6678 (4)	0.6589 (3)	0.0424 (8)	
O2	0.9184 (4)	0.7201 (4)	0.4586 (3)	0.0416 (8)	
O3	0.2725 (4)	0.5894 (4)	0.6746 (3)	0.0469 (8)	
O4	0.2232 (4)	0.6331 (4)	0.4730 (3)	0.0471 (8)	
N1	0.9757 (6)	0.3705 (5)	0.7847 (4)	0.0516 (8)	
C1	0.9135 (5)	0.7472 (5)	0.5736 (4)	0.0320 (9)	
C2	0.8510 (5)	0.8860 (5)	0.6110 (4)	0.0336 (10)	
H2	0.9467	0.9810	0.6299	0.040*	
C3	0.7803 (6)	0.8675 (6)	0.7305 (4)	0.0432 (11)	
H3A	0.8603	0.8430	0.7969	0.052*	
H3B	0.7635	0.9674	0.7597	0.052*	
C4	0.6161 (5)	0.7374 (6)	0.7083 (4)	0.0370 (10)	
H4A	0.6336	0.6357	0.6852	0.044*	
H4B	0.5746	0.7320	0.7859	0.044*	
C5	0.4889 (5)	0.7709 (5)	0.6026 (4)	0.0268 (8)	
H5	0.4777	0.8752	0.6296	0.032*	
C6	0.5562 (5)	0.7871 (5)	0.4806 (4)	0.0337 (10)	
H6A	0.5710	0.6866	0.4507	0.040*	
H6B	0.4761	0.8131	0.4152	0.040*	
C7	0.7222 (6)	0.9163 (5)	0.5037 (5)	0.0382 (10)	
H7A	0.7643	0.9202	0.4263	0.046*	
H7B	0.7049	1.0185	0.5252	0.046*	
C8	0.3170 (5)	0.6554 (5)	0.5811 (4)	0.0337 (10)	
C9	1.0536 (7)	0.2819 (7)	0.8583 (6)	0.0653 (17)	
H9	1.1446	0.2484	0.8461	0.078*	
C10	0.9687 (11)	0.2518 (9)	0.9557 (6)	0.104 (3)	
H10	0.9935	0.1930	1.0213	0.125*	
C11	0.8543 (7)	0.3871 (6)	0.8375 (5)	0.0512 (13)	
H11	0.7795	0.4418	0.8048	0.061*	
N2	0.8493 (6)	0.3188 (5)	0.9406 (4)	0.0516 (8)	
C12	0.7737 (19)	0.2877 (17)	1.0436 (13)	0.055 (3)	0.488 (9)
H12A	0.7196	0.3690	1.0584	0.066*	0.488 (9)
H12B	0.8591	0.2945	1.1188	0.066*	0.488 (9)
C13	0.6462 (19)	0.1259 (17)	1.0251 (14)	0.047 (3)	0.488 (9)
C14	0.653 (3)	0.034 (3)	1.120 (2)	0.064 (5)	0.488 (9)
H14	0.7379	0.0393	1.1893	0.077*	0.488 (9)
C15	0.516 (3)	0.076 (3)	0.9209 (18)	0.062 (4)	0.488 (9)
H15	0.5434	0.1302	0.8536	0.074*	0.488 (9)
C12'	0.6974 (18)	0.3329 (16)	1.0078 (12)	0.055 (3)	0.512 (9)
H12C	0.7410	0.3783	1.0960	0.066*	0.512 (9)
H12D	0.6391	0.4017	0.9648	0.066*	0.512 (9)
C13'	0.5796 (18)	0.1695 (16)	1.0001 (13)	0.047 (3)	0.512 (9)
C14'	0.598 (3)	0.076 (3)	1.093 (2)	0.064 (5)	0.512 (9)
H14'	0.6596	0.1367	1.1695	0.077*	0.512 (9)
C15'	0.456 (2)	0.110 (3)	0.8901 (17)	0.062 (4)	0.512 (9)
H15'	0.4158	0.1637	0.8254	0.074*	0.512 (9)

### Atomic displacement parameters (Å^2^)

**Table:**

	*U*^11^	*U*^22^	*U*^33^	*U*^12^	*U*^13^	*U*^23^
Co	0.0260 (3)	0.0332 (3)	0.0288 (3)	0.0076 (2)	0.0100 (2)	0.0080 (2)
O1	0.0416 (19)	0.0388 (17)	0.0489 (18)	0.0177 (15)	0.0047 (15)	0.0077 (15)
O2	0.045 (2)	0.0374 (17)	0.0500 (19)	0.0156 (15)	0.0224 (15)	0.0082 (14)
O3	0.0319 (18)	0.052 (2)	0.054 (2)	−0.0014 (15)	0.0210 (15)	0.0051 (16)
O4	0.0237 (17)	0.051 (2)	0.056 (2)	0.0035 (15)	−0.0051 (15)	0.0008 (16)
N1	0.056 (2)	0.0509 (18)	0.0342 (15)	−0.0122 (15)	0.0144 (14)	0.0067 (13)
C1	0.017 (2)	0.027 (2)	0.049 (3)	0.0003 (16)	0.0098 (18)	0.0050 (19)
C2	0.019 (2)	0.027 (2)	0.052 (3)	0.0010 (16)	0.0096 (18)	0.0020 (19)
C3	0.026 (2)	0.058 (3)	0.040 (2)	0.011 (2)	−0.0007 (19)	−0.010 (2)
C4	0.027 (2)	0.054 (3)	0.034 (2)	0.014 (2)	0.0094 (18)	0.015 (2)
C5	0.018 (2)	0.029 (2)	0.035 (2)	0.0073 (16)	0.0076 (16)	0.0060 (17)
C6	0.026 (2)	0.043 (2)	0.034 (2)	0.0116 (19)	0.0066 (17)	0.0079 (18)
C7	0.032 (2)	0.037 (2)	0.055 (3)	0.016 (2)	0.021 (2)	0.019 (2)
C8	0.026 (2)	0.031 (2)	0.047 (3)	0.0101 (18)	0.013 (2)	0.0025 (19)
C9	0.051 (3)	0.057 (3)	0.074 (4)	0.004 (3)	−0.012 (3)	0.035 (3)
C10	0.128 (7)	0.085 (5)	0.041 (3)	−0.046 (5)	−0.023 (4)	0.047 (3)
C11	0.052 (3)	0.051 (3)	0.048 (3)	−0.005 (2)	0.031 (2)	0.005 (2)
N2	0.056 (2)	0.0509 (18)	0.0342 (15)	−0.0122 (15)	0.0144 (14)	0.0067 (13)
C12	0.064 (9)	0.051 (6)	0.041 (6)	−0.012 (5)	0.034 (6)	−0.005 (4)
C13	0.056 (9)	0.040 (6)	0.044 (5)	−0.007 (4)	0.038 (6)	−0.006 (4)
C14	0.060 (13)	0.074 (12)	0.043 (8)	−0.014 (7)	0.021 (8)	−0.005 (7)
C15	0.071 (12)	0.067 (9)	0.040 (8)	−0.002 (7)	0.022 (7)	0.011 (5)
C12'	0.064 (9)	0.051 (6)	0.041 (6)	−0.012 (5)	0.034 (6)	−0.005 (4)
C13'	0.056 (9)	0.040 (6)	0.044 (5)	−0.007 (4)	0.038 (6)	−0.006 (4)
C14'	0.060 (13)	0.074 (12)	0.043 (8)	−0.014 (7)	0.021 (8)	−0.005 (7)
C15'	0.071 (12)	0.067 (9)	0.040 (8)	−0.002 (7)	0.022 (7)	0.011 (5)

### Geometric parameters (Å, °)

**Table:**

Co—O2^i^	2.008 (3)	C6—H6B	0.9700
Co—O3^ii^	2.035 (3)	C7—H7A	0.9700
Co—O1	2.044 (3)	C7—H7B	0.9700
Co—N1	2.044 (4)	C9—C10	1.388 (10)
Co—O4^iii^	2.117 (3)	C9—H9	0.9300
Co—Co^i^	2.7758 (10)	C10—N2	1.298 (11)
O1—C1	1.265 (5)	C10—H10	0.9300
O2—C1	1.260 (5)	C11—N2	1.303 (7)
O2—Co^i^	2.008 (3)	C11—H11	0.9300
O3—C8	1.268 (6)	N2—C12	1.395 (13)
O3—Co^iv^	2.035 (3)	N2—C12'	1.630 (14)
O4—C8	1.255 (5)	C12—C13	1.52 (2)
O4—Co^iii^	2.117 (3)	C12—H12A	0.9700
N1—C11	1.310 (7)	C12—H12B	0.9700
N1—C9	1.356 (7)	C13—C14	1.34 (3)
C1—C2	1.518 (6)	C13—C15	1.38 (2)
C2—C3	1.526 (6)	C14—C15^v^	1.47 (4)
C2—C7	1.528 (6)	C14—H14	0.9300
C2—H2	0.9800	C15—C14^v^	1.47 (4)
C3—C4	1.522 (6)	C15—H15	0.9300
C3—H3A	0.9700	C12'—C13'	1.507 (19)
C3—H3B	0.9700	C12'—H12C	0.9700
C4—C5	1.521 (5)	C12'—H12D	0.9700
C4—H4A	0.9700	C13'—C14'	1.35 (3)
C4—H4B	0.9700	C13'—C15'	1.40 (2)
C5—C8	1.513 (6)	C14'—C15'^v^	1.62 (3)
C5—C6	1.535 (6)	C14'—H14'	0.9300
C5—H5	0.9800	C15'—C14'^v^	1.62 (3)
C6—C7	1.529 (6)	C15'—H15'	0.9300
C6—H6A	0.9700		
			
O2^i^—Co—O3^ii^	91.45 (14)	C2—C7—C6	111.7 (3)
O2^i^—Co—O1	164.36 (13)	C2—C7—H7A	109.3
O3^ii^—Co—O1	90.51 (14)	C6—C7—H7A	109.3
O2^i^—Co—N1	98.07 (16)	C2—C7—H7B	109.3
O3^ii^—Co—N1	104.53 (16)	C6—C7—H7B	109.3
O1—Co—N1	96.44 (16)	H7A—C7—H7B	107.9
O2^i^—Co—O4^iii^	88.63 (14)	O4—C8—O3	123.1 (4)
O3^ii^—Co—O4^iii^	164.38 (14)	O4—C8—C5	118.6 (4)
O1—Co—O4^iii^	85.35 (13)	O3—C8—C5	118.3 (4)
N1—Co—O4^iii^	90.91 (16)	N1—C9—C10	105.5 (7)
O2^i^—Co—Co^i^	81.73 (9)	N1—C9—H9	127.3
O3^ii^—Co—Co^i^	97.00 (10)	C10—C9—H9	127.3
O1—Co—Co^i^	82.63 (9)	N2—C10—C9	109.2 (5)
N1—Co—Co^i^	158.46 (13)	N2—C10—H10	125.4
O4^iii^—Co—Co^i^	67.55 (10)	C9—C10—H10	125.4
C1—O1—Co	124.3 (3)	N2—C11—N1	112.8 (6)
C1—O2—Co^i^	127.3 (3)	N2—C11—H11	123.6
C8—O3—Co^iv^	109.5 (3)	N1—C11—H11	123.6
C8—O4—Co^iii^	142.2 (3)	C10—N2—C11	106.6 (5)
C11—N1—C9	105.9 (5)	C10—N2—C12	105.7 (9)
C11—N1—Co	124.5 (4)	C11—N2—C12	147.6 (9)
C9—N1—Co	129.6 (4)	C10—N2—C12'	138.7 (7)
O2—C1—O1	123.6 (4)	C11—N2—C12'	114.6 (7)
O2—C1—C2	117.6 (4)	C12—N2—C12'	33.2 (7)
O1—C1—C2	118.8 (4)	N2—C12—C13	113.8 (10)
C1—C2—C3	112.7 (4)	N2—C12—H12A	108.8
C1—C2—C7	112.7 (4)	C13—C12—H12A	108.8
C3—C2—C7	109.5 (4)	N2—C12—H12B	108.8
C1—C2—H2	107.2	C13—C12—H12B	108.8
C3—C2—H2	107.2	H12A—C12—H12B	107.7
C7—C2—H2	107.2	C14—C13—C15	118.9 (18)
C4—C3—C2	112.5 (4)	C14—C13—C12	118.3 (16)
C4—C3—H3A	109.1	C15—C13—C12	122.6 (15)
C2—C3—H3A	109.1	C13—C14—C15^v^	98.2 (16)
C4—C3—H3B	109.1	C13—C14—H14	130.9
C2—C3—H3B	109.1	C15^v^—C14—H14	130.9
H3A—C3—H3B	107.8	C13—C15—C14^v^	141.3 (19)
C5—C4—C3	110.3 (4)	C13—C15—H15	109.3
C5—C4—H4A	109.6	C14^v^—C15—H15	109.3
C3—C4—H4A	109.6	C13'—C12'—N2	108.8 (9)
C5—C4—H4B	109.6	C13'—C12'—H12C	109.9
C3—C4—H4B	109.6	N2—C12'—H12C	109.9
H4A—C4—H4B	108.1	C13'—C12'—H12D	109.9
C8—C5—C4	114.2 (3)	N2—C12'—H12D	109.9
C8—C5—C6	112.8 (3)	H12C—C12'—H12D	108.3
C4—C5—C6	110.2 (3)	C14'—C13'—C15'	119.8 (17)
C8—C5—H5	106.3	C14'—C13'—C12'	121.5 (15)
C4—C5—H5	106.3	C15'—C13'—C12'	118.6 (13)
C6—C5—H5	106.3	C13'—C14'—C15'^v^	138.1 (19)
C7—C6—C5	111.1 (3)	C13'—C14'—H14'	111.0
C7—C6—H6A	109.4	C15'^v^—C14'—H14'	111.0
C5—C6—H6A	109.4	C13'—C15'—C14'^v^	100.5 (13)
C7—C6—H6B	109.4	C13'—C15'—H15'	129.7
C5—C6—H6B	109.4	C14'^v^—C15'—H15'	129.7
H6A—C6—H6B	108.0		
			
O2^i^—Co—O1—C1	3.5 (7)	Co^iv^—O3—C8—O4	−5.3 (5)
O3^ii^—Co—O1—C1	100.7 (3)	Co^iv^—O3—C8—C5	172.4 (3)
N1—Co—O1—C1	−154.6 (3)	C4—C5—C8—O4	−153.7 (4)
O4^iii^—Co—O1—C1	−64.2 (3)	C6—C5—C8—O4	−26.8 (5)
Co^i^—Co—O1—C1	3.7 (3)	C4—C5—C8—O3	28.5 (5)
O2^i^—Co—N1—C11	−139.8 (4)	C6—C5—C8—O3	155.4 (4)
O3^ii^—Co—N1—C11	126.6 (4)	C11—N1—C9—C10	−0.6 (6)
O1—Co—N1—C11	34.4 (4)	Co—N1—C9—C10	179.1 (4)
O4^iii^—Co—N1—C11	−51.1 (4)	N1—C9—C10—N2	−0.2 (7)
Co^i^—Co—N1—C11	−51.9 (6)	C9—N1—C11—N2	1.2 (6)
O2^i^—Co—N1—C9	40.6 (5)	Co—N1—C11—N2	−178.5 (3)
O3^ii^—Co—N1—C9	−53.1 (5)	C9—C10—N2—C11	0.9 (7)
O1—Co—N1—C9	−145.3 (5)	C9—C10—N2—C12	−178.2 (7)
O4^iii^—Co—N1—C9	129.3 (5)	C9—C10—N2—C12'	177.2 (8)
Co^i^—Co—N1—C9	128.5 (5)	N1—C11—N2—C10	−1.3 (6)
Co^i^—O2—C1—O1	8.4 (6)	N1—C11—N2—C12	177.0 (12)
Co^i^—O2—C1—C2	−173.4 (3)	N1—C11—N2—C12'	−178.7 (6)
Co—O1—C1—O2	−8.0 (6)	C10—N2—C12—C13	−83.2 (12)
Co—O1—C1—C2	173.8 (3)	C11—N2—C12—C13	98.5 (15)
O2—C1—C2—C3	155.3 (4)	C12'—N2—C12—C13	91.3 (19)
O1—C1—C2—C3	−26.4 (5)	N2—C12—C13—C14	132.3 (16)
O2—C1—C2—C7	30.8 (5)	N2—C12—C13—C15	−54 (2)
O1—C1—C2—C7	−150.9 (4)	C15—C13—C14—C15^v^	−12 (3)
C1—C2—C3—C4	−70.3 (5)	C12—C13—C14—C15^v^	162.6 (15)
C7—C2—C3—C4	56.0 (5)	C14—C13—C15—C14^v^	18 (4)
C2—C3—C4—C5	−57.7 (5)	C12—C13—C15—C14^v^	−155 (3)
C3—C4—C5—C8	−175.0 (3)	C10—N2—C12'—C13'	−62.0 (14)
C3—C4—C5—C6	56.8 (5)	C11—N2—C12'—C13'	114.1 (10)
C8—C5—C6—C7	174.5 (3)	C12—N2—C12'—C13'	−70.1 (16)
C4—C5—C6—C7	−56.6 (5)	N2—C12'—C13'—C14'	90.6 (17)
C1—C2—C7—C6	71.5 (5)	N2—C12'—C13'—C15'	−85.0 (15)
C3—C2—C7—C6	−54.8 (5)	C15'—C13'—C14'—C15'^v^	18 (4)
C5—C6—C7—C2	56.1 (5)	C12'—C13'—C14'—C15'^v^	−158 (2)
Co^iii^—O4—C8—O3	12.1 (8)	C14'—C13'—C15'—C14'^v^	−12 (3)
Co^iii^—O4—C8—C5	−165.6 (3)	C12'—C13'—C15'—C14'^v^	163.8 (13)

Symmetry codes: (i) −*x*+2, −*y*+1, −*z*+1; (ii) *x*+1, *y*, *z*; (iii) −*x*+1, −*y*+1, −*z*+1; (iv) *x*−1, *y*, *z*; (v) −*x*+1, −*y*, −*z*+2.
